# Thermodynamics and Kinetics of Electron Transfer of Electrode-Immobilized Small Laccase from *Streptomyces coelicolor*

**DOI:** 10.3390/molecules27228079

**Published:** 2022-11-21

**Authors:** Giulia Di Rocco, Gianantonio Battistuzzi, Antonio Ranieri, Carlo Augusto Bortolotti, Marco Borsari, Marco Sola

**Affiliations:** 1 Department of Life Sciences, University of Modena and Reggio Emilia, Via Campi 103, 41125 Modena, Italy; 2 Department of Chemical and Geological Sciences, University of Modena and Reggio Emilia, Via Campi 103, 41125 Modena, Italy

**Keywords:** small laccase, electron transfer, protein voltammetry, self-assembled monolayers, redox thermodynamics, protein-electrode linkage

## Abstract

The thermodynamic and kinetic properties for heterogeneous electron transfer (ET) were measured for the electrode-immobilized small laccase (SLAC) from *Streptomyces coelicolor* subjected to different electrostatic and covalent protein-electrode linkages, using cyclic voltammetry. Once immobilized electrostatically onto a gold electrode using mixed carboxyl- and hydroxy-terminated alkane-thiolate SAMs or covalently exploiting the same SAM subjected to N-hydroxysuccinimide+1-Ethyl-3-(3-dimethylaminopropyl)carbodiimide (NHS-EDC) chemistry, the SLAC-electrode electron flow occurs through the T1 center. The E°′ values (from +0.2 to +0.1 V vs. SHE at pH 7.0) are lower by more than 0.2 V compared to the protein either in solution or immobilized with different anchoring strategies using uncharged SAMs. For the present electrostatic and covalent binding, this effect can, respectively, be ascribed to the negative charge of the SAM surfaces and to deletion of the positive charge of Lys/Arg residues due to amide bond formation which both selectively stabilize the more positively charged oxidized SLAC. Observation of enthalpy/entropy compensation within the series indicates that the immobilized proteins experience different reduction-induced solvent reorganization effects. The E°′ values for the covalently attached SLAC are sensitive to three acid base equilibria, with apparent pK_a_ values of pK_a1ox_ = 5.1, pK_a1red_ = 7.5, pK_a2ox_ = 8.4, pK_a2red_ = 10.9, pK_a2ox_ = 8.9, pK_a2red_ = 11.3 possibly involving one residue close to the T1 center and two residues (Lys and/or Arg) along with moderate protein unfolding, respectively. Therefore, the E°′ value of immobilized SLAC turns out to be particularly sensitive to the anchoring mode and medium conditions.

## 1. Introduction

**Figure 1:**
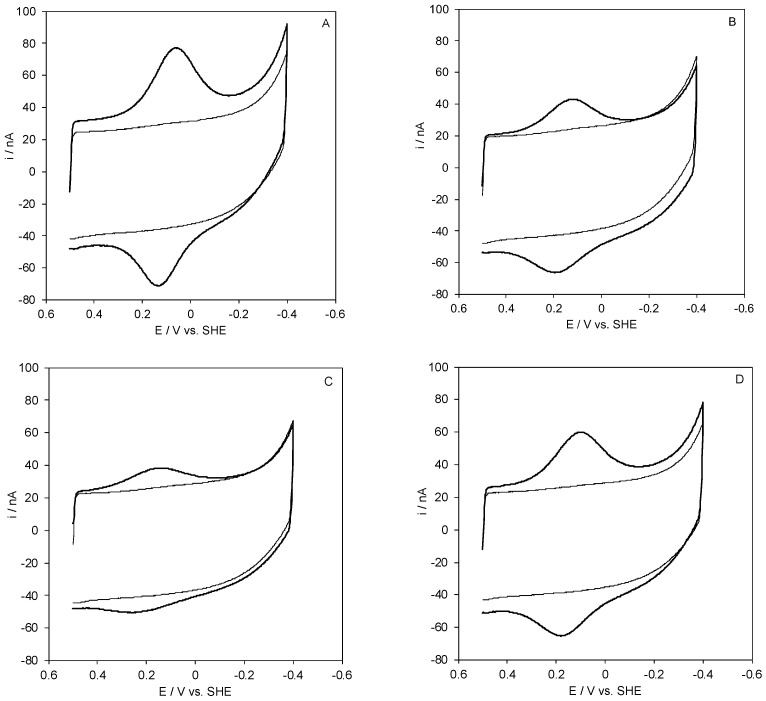
Cyclic voltammograms for wt *Streptomyces coelicolor* small laccase (SLAC) electrostatically bound on a polycrystalline gold electrode coated with a SAM of MUA (**A**), 1:1 MUA/MU (**B**), 1:2 MUA/MU (**C**), and 1:3 MUA/MU (**D**). CVs were recorded in 5 mM Tris-HCl buffer, 5 mM sodium perchlorate, pH 7. Scan rate: 0.05 V s^−1^, T = 20 °C.

Multicopper blue oxidases (BMCOs) from plants, fungi, and animals, such as laccase, bilirubin oxidase, ascorbate oxidase, and ceruloplasmin, catalyze the oxidation of a variety of organic substrates by molecular oxygen which is reduced to water [1,2,3,4]. BMCOs contain four copper centers: a mononuclear Type-1 (T1) center and a composite mononuclear Type-2 (T2) and dinuclear Type-3 (T3) center yielding a trinuclear (T2/T3) copper cluster. These enzymes couple four one-electron substrate oxidation steps occurring at the T1 center to a two-electron reduction of molecular oxygen to water carried out by the T2/T3 cluster (Figure 1) [4,5,6,7]. Laccase-like BMCOs from bacterial sources were identified [8,9,10] and extensively investigated with particular reference to the redox properties and the mechanism of the four-electron reaction [11,12,13,14,15,16,17]. Bacterial BMCOs show little sequence identity and different kinetic properties compared with fungal and plant analogues. The oxidation rates are usually slow, although one of them, the copper efflux oxidase (CueO) from *Escherichia coli*, was reported [18] to electrocatalyze dioxygen reduction about nine times more efficiently than laccase [19].

Laccases are largely exploited for the bio-electroreduction of dioxygen in the cathode of biofuel cells and oxygen-sensitive biosensors [20,21,22,23]. This is also thanks to intensive fundamental bioelectrochemical investigation of direct heterogeneous electron transfer (ET) between the protein and solid electrodes [18,24,25,26,27,28,29,30,31,32,33,34,35]. In such electrochemical environment, electrons pass directly from the electrode surface to the substrate oxidizing T1 copper center of the enzyme. However, efficient catalysis can be obtained provided the enzyme be properly oriented to facilitate heterogeneous ET, namely with the T1 site pointing toward the electrode surface [26,33,34,36,37,38,39,40]. Enzyme efficiency and stability also constitute an issue for the development of bioelectronic devices. In this respect, bacterial laccases constitute a resource. In fact, heterologous expression of recombinant proteins in bacteria is viable and the resulting proteins are non-glycosylated, which may facilitate electron transfer. Moreover, these species feature an enhanced thermostability and activity in a wider pH range and a larger stability under denaturing conditions [41,42] compared to fungal laccases. In particular, the small laccase (SLAC) from *Streptomyces coelicolor* was previously reported to be highly active at pH 7, showing the same specificity toward dioxygen reduction and non-specificity toward the reducing substrate as other laccases, making it an ideal candidate for enzyme bioelectrocatalysis [41]. The heterogeneous ET of electrode-immobilized SLAC has been investigated to some extent along with the catalytic activity against various substrates [43,44,45]. In this work, we have studied how the thermodynamic and kinetic features of the SLAC-electrode electron flow are affected by the features of electrode functionalization using protein voltammetry. In particular, we focused on the nature of the self-assembled monolayer (SAM) covering the solid electrode and the type of protein–electrode linkage, either electrostatic or covalent, to be compared with previous studies made with uncharged SAMs. The kinetics and thermodynamics of ET were also measured along with their pH dependence. We found that these parameters are remarkably sensitive to the electrostatics, conformational changes, and solvent reorganization effects at the SAM-SLAC interface. This study extends the knowledge on the ability of SLAC to talk with solid electrodes under different conditions that may help enlarging its potential range of practical or industrial applications.

## 2. Results and Discussion

### 2.1. Voltammetric Responses of Electrode-Immobilized SLAC

SLAC yields a stable, well defined electron transfer process upon electrostatic immobilization on a polycrystalline gold wire coated with COOH-terminated SAMs, taking advantage of its positive charge at neutral pH (pI = 8.2 ± 0.2 and 7.3 ± 0.2 for the fully oxidized and fully reduced state, respectively). In fact, COOH-terminated SAMs are characterized by local pK_a_ values of about 5–5.5 [46,47], therefore, at neutral pH the SAM surface is negatively charged. Reasonably well-shaped voltametric responses were obtained using SAMs of 11-mercapto-1-undecanoic acid (MUA hereafter) and mixed SAMs of 11-mercapto-1-undecanoic acid/11-mercapto-1-undecanol (MUA/MU hereafter) in the 3:1, 1:1, and 1:2 ratios (Figure 1).

**Figure 2:**
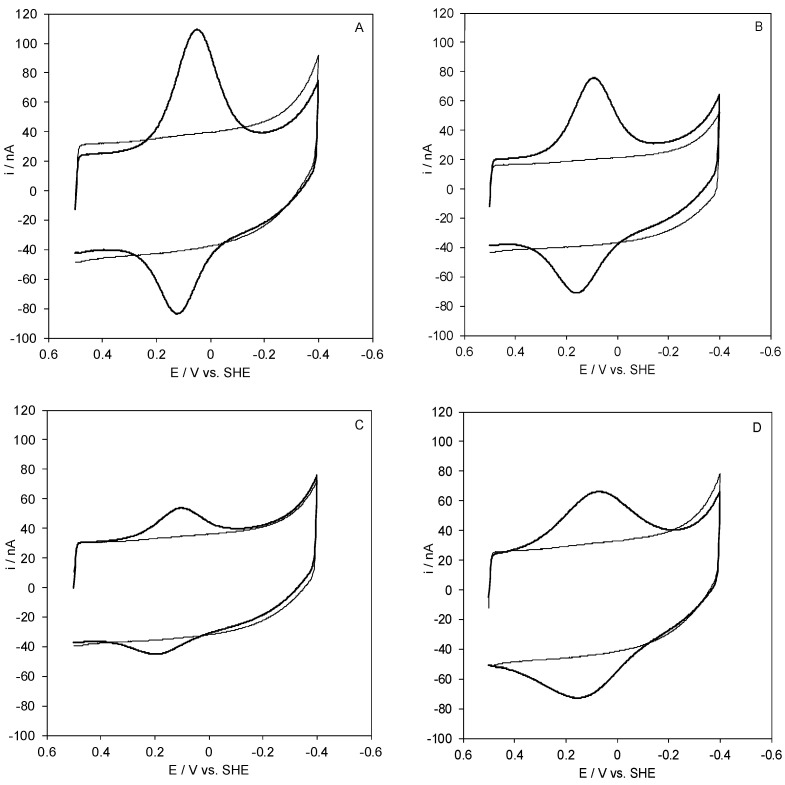
Cyclic voltammograms for wt *Streptomyces coelicolor* small laccase (SLAC) covalently bound to a SAM of MUA (**A**), 1:1 MUA/MU (**B**), 1:2 MUA/MU (**C**), and 1:3 MUA/MU (**D**) through NHS/EDC chemistry. CVs were recorded in 5 mM Tris-HCl buffer, 5 mM sodium perchlorate, pH 7. Scan rate: 0.05 V s^−1^, T = 20 °C.

Similarly, good CV responses were obtained for SLAC that was covalently immobilized on the same SAMs through NHS-EDC linkage (Figure 2).

**Figure 3:**
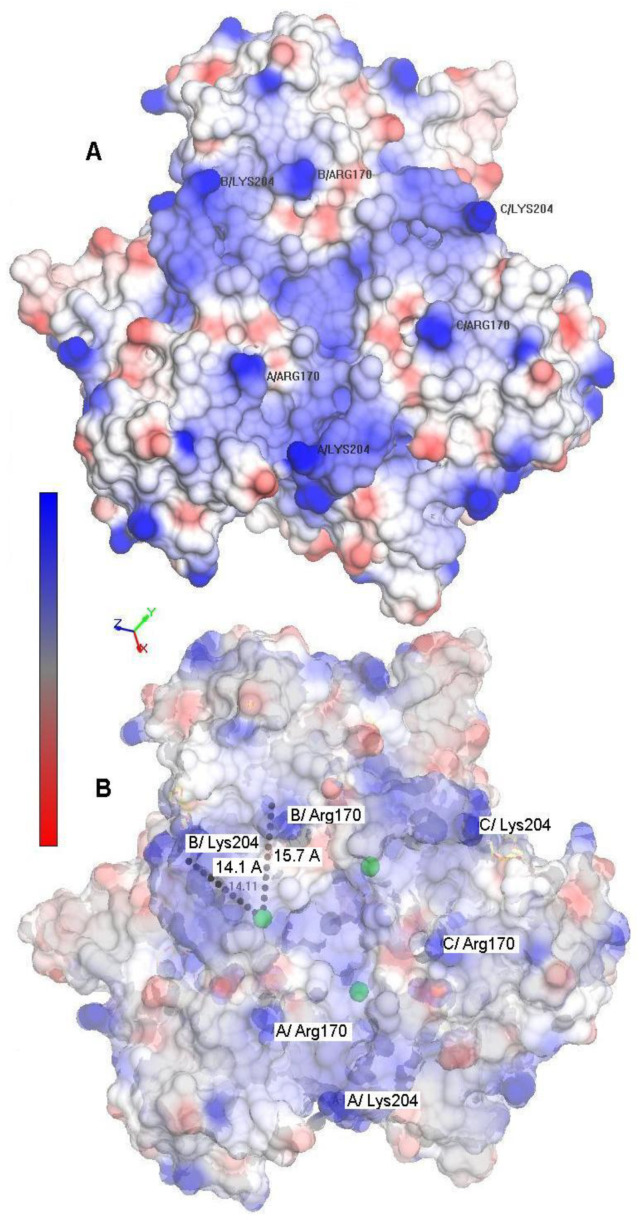
(**A**) Surface electrostatic potential of the homo-dimeric *Streptomyces coelicolor* small laccase (SLAC) showing neutral (white), positively (blue) and negatively (red) charged areas. The three monomers are labeled A, B, and C. The figure was prepared using the NOC 3.01 package from the X-ray structure (PDB code 3CG8) [42]. (**B**) Schematic representation of SLAC with a decreased opacity. The T1 copper ions are depicted as green dots. The Arg170 and Lys204 residues for all subunits are highlighted. The distances form the T1 copper center to the selected residues of subunit B are calculated from the crystallographic structure.

Invariably, currents were found to increase linearly with the scan rate, as expected for diffusionless electrochemical processes. The ratio between the anodic and cathodic peak areas was found to be about one and peak–peak separation values (ΔE_p_) at 50 mV s^−1^ ranged from 45 to 95 mV, indicating a quasi-reversible electrochemical behavior of the immobilized enzyme. These responses can be attributed to the Cu(II)/Cu(I) redox couple of the T1 site, due to the absence of any electrochemical response under the same conditions for the T1-depleted form of the enzyme (T1D SLAC) in which the T1 Cu-coordinating Cys was substituted with Ser (C288S) resulting in an empty T1 site and an intact trinuclear cluster (TNC) [48]. The reduction potential (E°′) values are listed in Table 1. The crystal structure of SLAC (PDB code—3CG8 [42]) was explored to evaluate possible protein–SAM binding sites. Figure 3 shows the calculated electrostatic potential of the enzyme surface at pH 7.

In each subunit, a significantly dense patch of positively charged (blue) amino acid residues is located just above the T1 copper center, which is located about 6–8 Å below the protein surface. Lys204 and Arg170 protrude from this patch. Therefore, these positively charged residues most likely interact with the negatively charged surface that is formed by the carboxylate groups of MUA, yielding an efficient electron tunneling route. The distances of these residues from the T1 copper center are consistent with this hypothesis. Lys204 and Arg170 are also the most likely candidates to yield the covalent linkage of the protein with the SAM surface with EDC/NHS linkage.

**Figure 4:**
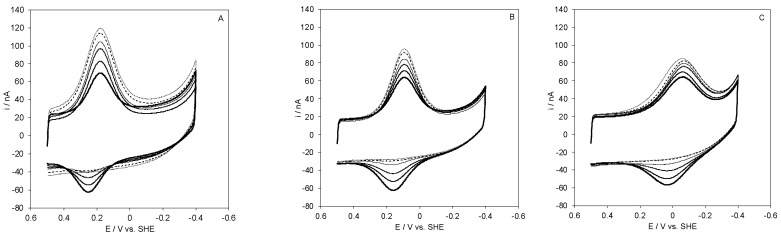
Cyclic voltammograms for SLAC covalently immobilized on MUA/MU SAM at different exposure times of the electrochemical cell (initially under argon) to air at normal atmospheric pressure. CVs were recorded in 5 mM phosphate buffer, 5 mM sodium perchlorate at pH 4.5 (**A**), 7.0 (**B**), and 9.6 (**C**). Scan rate: 0.05 V s^−1^, T = 20 °C.

The SLAC surface coverages (Γ_0_) for the electrostatic protein immobilization decreases with decreasing MUA content of the SAM (Table 1), namely in the order: MUA > MUA/MU 3:1 > MUA/MU 1:1 > MUA/MU 1:2, hence with decreasing the negative charge density of the SAM surface (Table 1). In parallel, the CV peak currents decrease in the same order. Both effects are clearly the result of the decreased electrostatic SAM/protein interaction. The surface coverage follows the same order for covalent protein immobilization using the EDC/NHS chemistry (Table 1) because the coverage is determined by the first step of protein–SAM interaction, which is electrostatic and precedes ester bond formation. In all cases, the amounts of immobilized electroactive protein are much lower than those that were obtained with other procedures [49,50] possibly due to unfolding effects induced by the SAM surface or, more likely, to the presence of several positively charged regions on the protein surface, responsible for the protein–SAM attachment, of which only a few allow for efficient ET. No appreciable changes in the electrochemical responses for SLAC on the various functionalized electrodes were observed between 5 and 45 °C. For the protein covalently immobilized on a MUA/MU-coated gold electrode, CVs that were recorded at different pH values and increasing exposure to atmospheric dioxygen are shown in Figure 4.

The cathodic (anodic) peak currents progressively increase (decrease) with increasing the exposure time. This is a typical catalytic behavior and indicate that the adsorbed protein is still able to catalyze O_2_ reduction. Complete removal of dioxygen restored the pristine CV signal of SLAC. The catalytic currents could then be re-obtained upon dioxygen addition. Therefore, at all the investigated pH values, the protein layer immobilized on MUA/MU is stable and re-usable. The differences that were observed in the evolution of the CV curves over time at the three pH values are small which tell us that the catalytic activity is comparable.

### 2.2. Reduction Thermodynamics and pH-Induced Changes

The E°′ values of the electrostatically-immobilized SLAC at neutral pH (from +0.097 to +0.199 V) are significantly lower than the values that were reported previously for the protein in solution (+0.50 V [41] and +0.375 V [51]) and immobilized either on pyrene- and neocuproine-modified graphite disc electrodes (+0.37 and +0.39 V, respectively [44]) or carbon nanotube-modified glassy carbon electrodes (+0.43 V [45]). Such remarkable stabilization of the oxidized form of the enzyme by the negatively charged SAM can be justified on simple electrostatic grounds due to the stabilization of the more positively charged cupric state. Accordingly, the E°′ values decrease with increasing the negative surface charge density of the various SAMs (Table 1). Such electrostatic immobilization-induced E°′ decrease is much greater than that which was observed for cytochrome *c* under similar conditions [52,53], but similar to that which was observed for plastocyanin [53,54]. This suggests a particularly intense electrostatic effect, perhaps to be related to a closer proximity of the T1 center to the surface of the SAM under adsorption conditions.

**Figure 5:**
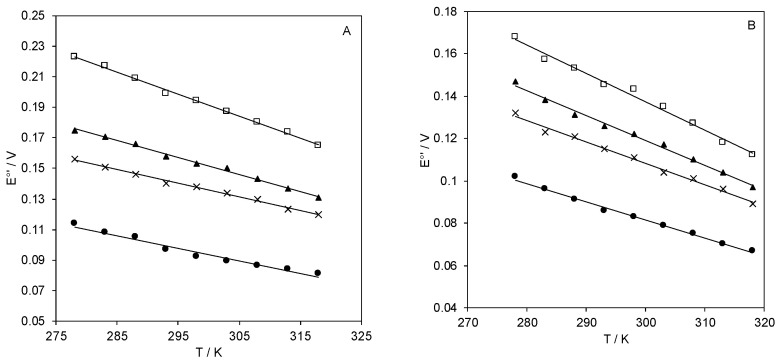
E°′ vs. T plots of *Streptomyces coelicolor* small laccase (SLAC) immobilized on a gold electrode coated with electrostatically (**A**) MUA (●), 3:1 MUA/MU (×), 1:1 MUA/MU (▲), 1:2 MUA/MU (□), or covalently (**B**) MUA(EDC/NHS) (●), 3:1 MUA/MU (EDC/NHS) (×), 1:1 MUA/MU (EDC/NHS) (▲), 1:2 MUA/MU (EDC/NHS) (□), respectively. pH = 7.0. Reduction entropy (ΔS°′) is calculated from the slope of the linear regression lines. The error bars are not shown because they have the same dimension of the symbols.

The temperature dependence of E°′ for the immobilized SLAC invariably show a monotonic linear decrease with increasing temperature in the range of 5–40 °C (Figure 5).

**Figure 6:**
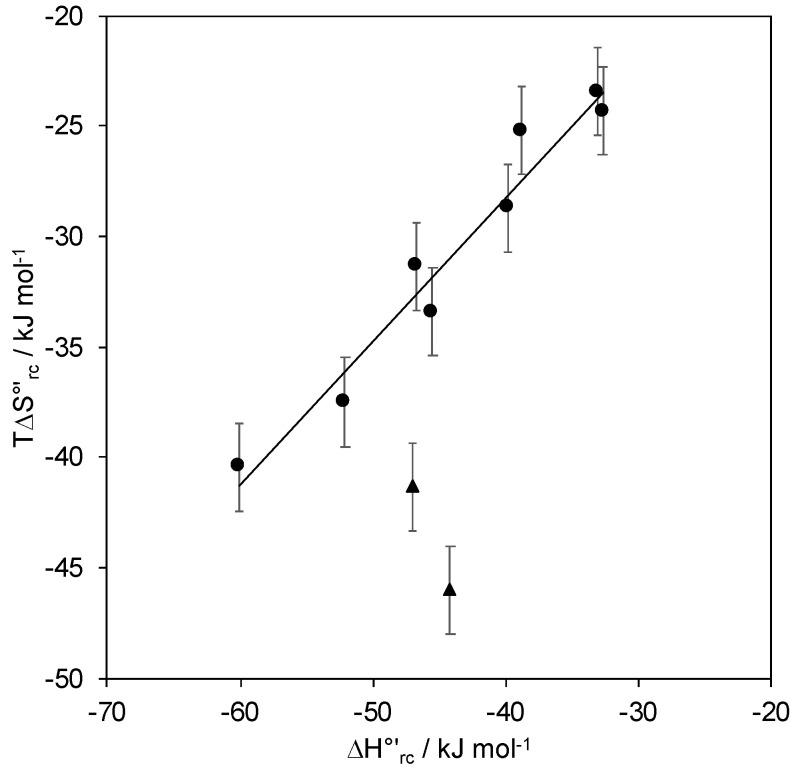
Enthalpy–entropy compensation plots for the reduction thermodynamics of *Streptomyces coelicolor* small laccase (SLAC) electrostatically and covalently immobilized on a gold electrode coated with MUA, 3:1 MUA/MU, 1:1 MUA/MU, 1:2 MUA/MU, MUA(EDC/NHS), 1:1 MUA/MU(EDC/NHS) at pH 7.0 (●), and covalently immobilized with 1:1 MUA/MU(EDC/NHS) at pH 8.4 and 9.6 (▲). T=293 K. Error bars are reported when available or have the same dimensions of symbols.

The thermodynamics for protein reduction are listed in Table 1. The decrease in E°′ due to the electrostatic interaction with the negative SAM is indeed totally enthalpic in origin. In fact, the reduction enthalpies become gradually less negative (indicating a progressive stabilization of the oxidized state, reasonably of electrostatic origin) with increasing the negative charge density of the SAM (namely in the order: 1:2 MUA/MU, 1:1 MUA/MU, 3:1 MUA/MU, MUA), while the entropic term yields an opposite effect on E°′. This is mainly due to solvent reorganization effects around the protein following the electron transfer process, as the changes in charge distribution modify the H-bonding network within the hydration sphere of the protein. These opposite (compensatory) enthalpy/entropy changes are indicative of differences in reduction-induced solvent reorganization effects for SLAC immobilized on the various SAMS [53,55,56,57,58]. This is shown in the compensation plot of Figure 6 which slows a linear regression indicating a certain degree of H-S compensation which results in free energy changes lower than the variations in the individual enthalpic and entropic terms.

The covalent immobilization of SLAC on MUA, 3:1 MUA/MU, 1:1 MUA/MU, and 1:2 MUA/MU yields a comparable E°′ decrease with respect to the above cases of electrostatic binding, which is again entirely due to the enthalpic contribution. This effect can be attributed to the deletion of the positive charge of Lys/Arg residues due to amide bond formation. The fact that the thermodynamic contributions are similar for the two SAM-protein constructs suggests that the protein orientation toward the electrode (that affects mainly ΔH°’_rc_) and reduction-induced solvent reorganization effects (determining ΔS°′_rc_) are conserved and, therefore, that the residues that are involved in protein binding (possibly Lys204 and Arg170) are likely the same. This is also supported by the fact that the four data points for covalently bound SLAC and those corresponding to the electrostatic attachment follow the same H-S compensation plot (Figure 6).

The reduction thermodynamics at pH 7, compared with those for other T1 centers in laccases and cupredoxins indicate that the positive E°′ value for these centers invariably have an enthalpic origin as both enthalpic and entropic terms are negative, although the latter term may be significant, as for SLAC (Table 2). The nature of the adsorbing surface certainly affects E°′, but the compensative nature of the two thermodynamic terms is conserved. The E°′ value of SLAC immobilized on MUA/MU is low compared to other copper proteins because of remarkable negative ΔS°′ values. Indeed, the ΔH°’ values are comparable with those of other Type 1 Cu centers with much more positive potentials.

**Figure 7:**
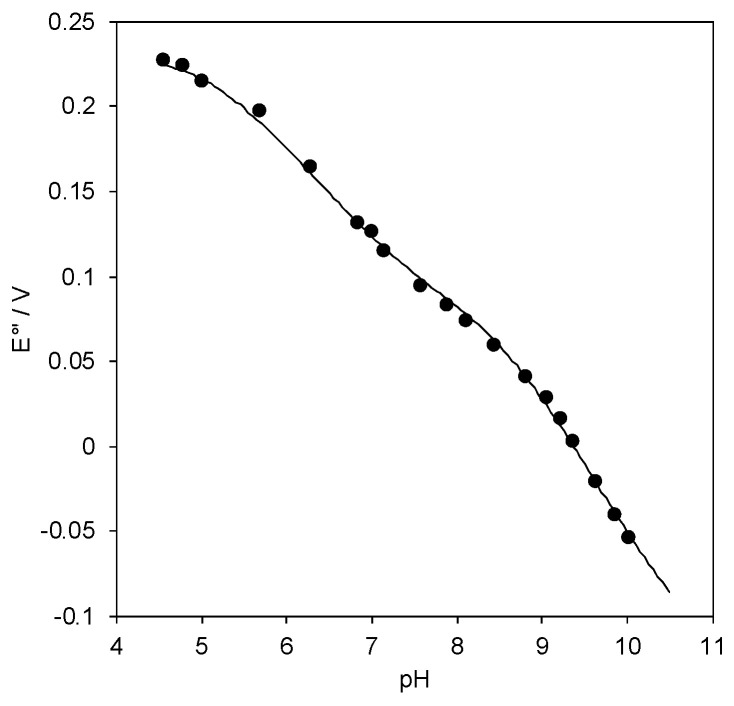
pH dependence of E°′ for *Streptomyces coelicolor* small laccase (SLAC) covalently bound to 1:1 MUA/MU gold electrode through EDC/NHS linkage procedure. 5 mM Tris-HCl buffer plus 5 mM sodium perchlorate, T = 20 °C. The error bars are not shown because they have the same dimension of the symbols.

The pH dependence of E°′ for SLAC covalently bound to 1:1 MUA/MU-functionalized gold electrode through the EDC/NHS linkage procedure is shown in Figure 7. The reduction thermodynamics at the selected pH values of 7.0, 8.4, and 9.6 are listed in Table 1. The E°′ vs. pH plot exhibits two linear regions, the former between pH 5.7 and 7.9 and the latter starting at about pH 9, with a slope of 58 and 85 mV per pH unit, respectively, corresponding to the loss of about one and two protons with small pK_a_ differences. The pH profile and the overall E°′ change at the two extremes of pH is consistent with that which was observed previously for the same species immobilized on pyrene- and neocuproine-modified graphite disc electrodes [44]. The data were fitted to a three-equilibria equation involving one and two reduction induced proton uptake at low and high pH, respectively:

(1) E°′=E°′low pH+0.059·log[(Ka1Ox+[H+])(Ka1Red+[H+])]+0.059·log[(Ka2Ox+[H+])(Ka2Red+[H+])]+0.059·log[(Ka3Ox+[H+])(Ka3Red+[H+])]

where E°′ is the formal potential at any given pH, E°′_low pH_ is the limit formal potential at acid pH, K_a1Ox_, Ka_2Ox_, and K_a3Ox_ are the acid-base equilibrium constants for the oxidized protein, while K_a1Red_, Ka_2Red_, and K_a3Red_ are the acid-base equilibrium constants for the reduced protein.

The data fit is satisfactory and the pK_a_ values are the following: pK_a1ox_ = 5.1, pK_a1red_ = 7.5, pK_a2ox_ = 8.4, pK_a2red_ = 10.9, pK_a2ox_ = 8.9, and pK_a2red_ = 11.3. It is likely that in the first equilibrium the oxidized enzyme undergoes reduction followed by proton uptake due to an oxidation state-dependent residue deprotonation in the surroundings of the metal center. At pH values above 9, a two-proton loss from Lys or Arg residues likely occurs accompanied by partial protein unfolding. The latter appears not to be dramatic as the protein maintains good electroactivity. However, the reduction thermodynamics that were measured at pH 8.4 and 9.6 (Table 1) suggest that pH-induced protein changes do occur under these conditions, as in the H-S compensation plot (Figure 6); both data points at pH 8.4 and 9.6 are clear outliers. If the changes in the thermodynamic terms had been determined only (or predominantly) by solvent reorganization effects, no or little change would be expected in the reduction free energy and the data would fall into the linear regression. These as yet unidentified residues must be responsible also for the pH-dependent catalytic activity of SLAC in solution which is indeed controlled by two equilibria with pK_a_ values of 7.3 and 9.2 [41].

### 2.3. Kinetics of Heterogeneous Protein-Electrode ET

**Figure 8:**
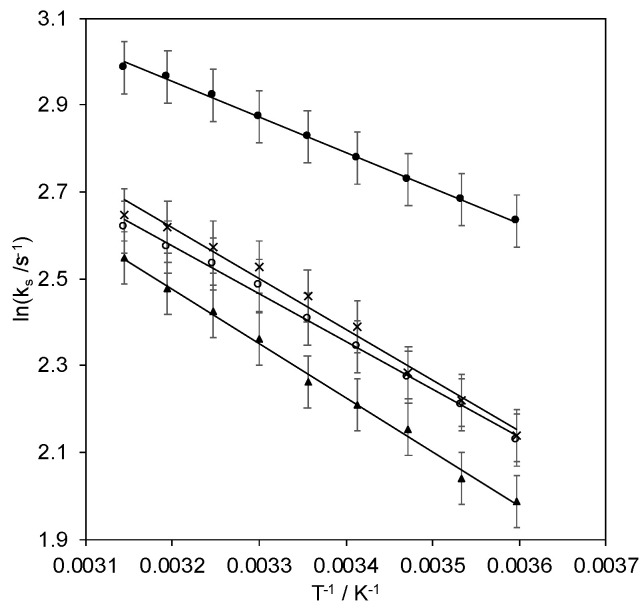
ln *k_s_* vs. 1/T plots of *Streptomyces coelicolor* small laccase (SLAC) covalently bound to 1:1 MUA/MU gold electrode through EDC/NHS linkage procedure at different pH values: pH = 7.0 (○), pH = 8.4 (●), pH = 9.6 (▲), and electrostatically immobilized on 1:1 MUA/MU gold electrode at pH = 7.0 (×). Activation enthalpies (ΔH^#^) are calculated from the slope of the linear regression curves.

The rate constants, *k*_s_, for the ET process between the adsorbed protein and the electrode that were determined at different pH values using the Laviron’s model [63] are listed in Table 3, along with the activation enthalpies that were obtained from the Arrhenius equation, namely from the ln *k_s_* vs. 1/T plots (Figure 8). These *k*_s_ values are very similar to those that were determined previously for SLAC that was adsorbed on pyrene- and neocuproine-modified graphite disc electrodes of about 1 s^−1^ [44], indicating that the present electrostatic and covalent SAM–protein interactions have the same efficiency as the hydrophobic ones in providing a viable route for intermolecular ET [44]. At pH 8.43, the electron transfer is most efficient. This pH value falls exactly in the plateau region between the two equilibria in the pH profile of E°′ and also corresponds to the condition of maximum catalytic activity [41] and is close to the protein isoelectric point (8.2). Thus, this charge condition allows for the maximum electron transfer ability which favors the catalytic activity. Since the entropic term of the free activation energy is considered negligible in several electron transfer proteins [64,65,66], the ΔH^#^ values that were obtained by the Arrhenius equation can be considered approximately equal to ΔG^#^. Therefore, the reorganization energy of the heterogeneous ET, λ, can be evaluated from the Marcus equation as λ = 4 ΔG^#^ = 4 ΔH^#^ (Table 3).

At pH 8.43, the activation enthalpy reaches a minimum and this pH value protein reduction is no longer coupled to a proton uptake. This induces a decrease of the reorganization energy and facilitates protein reduction compared to pH 7.0 and 9.6. In the latter case, the increase in the activation enthalpy can reasonably be attributed, besides to the involvement of a proton loss in the reduction mechanism, also to a pH-induced unfolding process.

The distance of ET between the T1 Cu center and electrode surface can probably be evaluated from the Marcus equation:

(2) ks=ν0·e[−β·(r−r0)]·e[−ΔG#R·T]

where ν_0_ is kT/h = 6∙10^12^ s^−1^ (h is the Planck constant, *k* the Boltzman constant), *β* ist the tunnelling constant, (*r* − *r_0_*) is the tunnelling distance, *r* is the effective distance between the electrode surface and the electron transfer center in the protein (the T1 Cu center in our case), and *r_0_* is the minimum distance between the electron transfer centers (in general the sum of the van der Waals radii of the involved redox centers) [64,67,68]. Neglecting ΔS^#^:

(3) ks=ν0·e[−β·(r−r0)]·e[−ΔH#R·T]

namely:

(4) lnks=lnν0+[−β·(r−r0)]−ΔH#R·T

A *β* value of 1 Å^−1^ is usually used for the electron tunneling through the covalently bound chain of alkane-thiolate carboxylic acid and protein matrix and r_0_ reasonably ranges between 0 and 3 Å [54,64,65,69,70,71,72].

The lower and upper limit values for the tunneling distance at pH 7.0, 8.4, and 9.6 Å for the covalently electrode-immobilized SLAC that were estimated from the plot of ln*k_s_* vs. ΔH^#^/(RT) at the various temperatures are reported in Table 3 together with the values for the electrostatically immobilized protein at pH 7. As the tunneling distance across the MUA chain is about 19 Å [73,74], an estimate of the distance between the SAM surface and the T1 copper ion ranging from 1.3 to 4.3, from 1.8 to 4.8, and from 1.1 to 4.1 Å is obtained at pH 7.0, 8.4, and 9.6, respectively, for the covalently linked protein, while an estimate ranging from 1.1 to 4.1 Å of 1.1–4.1 Å is obtained for the electrostatic immobilization. These values are very similar, suggesting that the geometry of the protein covalently or electrostatically immobilized, also at different pH values, is the same, which is in agreement with the above consideration on the reduction thermodynamics. However, these values are lower than that which was calculated from the crystal structure by assuming the ET occurring through Lys204 or Arg170. This discrepancy could be explained by considering that proteins and SAMs are able to undergo relevant structural changes during the interaction, resulting in a tighter adsorption geometry than that predicted from the crystallographic data. In particular, in this case, the presence of a cavity on the protein surface just above the T1 ion could allow the carboxylic heads of MUA to enter the cavity, thereby positioning at a shorter distance to the T1 center. An approach based on this scheme was used by Armstrong et al. [75] and Chidsey et al. [76] to establish an efficient communication between enzymes (*Tv*L and cytochrome c oxidase, respectively) and electrode using the so called ‘wired’ or ‘click-chemistry’. These second generation-SAMs are able to yield significantly electron transfer constant and catalytic activity.

## 3. Experimental Section

### 3.1. Materials

The small laccase (SLAC) from *Streptomyces coelicolor* variant was kindly donated by Prof. G. W. Canters of the Leiden University (NL) [48]. All chemicals were reagent grade. 11-mercapto-1-undecanoic acid (MUA) and 11-mercapto-1-undecanol (MU) were purchased from Sigma-Aldrich. Water was purified through a Milli-Q Plus Ultrapure Water System coupled with an Elix-5 Kit (Millipore, Burlington, MA, USA).

### 3.2. Electrochemical Measurements

**Scheme 1:**
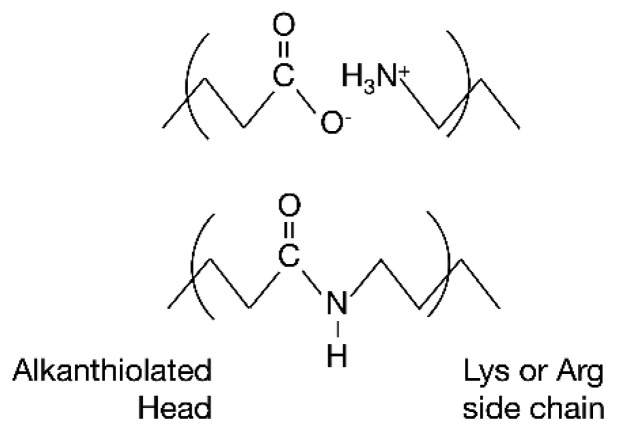
Schematic representation of the different strategies that were employed for SLAC immobilization. Upper panel: electrostatic interaction. Lower panel: covalent immobilization via formation of amide bond.

Cyclic voltammetry (CV) experiments were performed in a cell for small volume samples (0.5 mL) under argon using a potentiostat/galvanostat mod. 273A (EG&G PAR, Oak Ridge, TN, USA). A 1 mm-diameter polycrystalline gold wire, a platinum sheet, and a Standard Calomel Electrode (SCE) were used as a working, counter, and reference electrode, respectively. A stepwise treatment with concentrated H_2_SO_4_, flaming, concentrated H_2_SO_4_, concentrated KOH, 15 electrochemical cycles from −0.4 V to +1.5 V vs. SCE in 1 M H_2_SO_4_, and a final rinsing with purified water was adopted to clean the surface of the working electrode. All the applied potentials and E°′ values that were reported in this work were referred to the standard hydrogen electrode (SHE), unless otherwise specified. MUA or mixed MUA-MU SAM coating of the working gold wire was obtained by dipping the polished gold electrode into a 1 mM ethanol solution of MUA or both MUA and MU for 12 h and then rinsing it with water. 10 voltammetric cycles from +0.2 V to −0.4 V in a 0.1 M sodium perchlorate solution (outgassed with argon) were then performed to improve the structural organization of the monolayer on the gold surface and verify the effectiveness of the cleaning procedure. The resulting CV was taken as the background and checked for the absence of spurious signals. Electrostatic protein adsorption on the SAM-coated Au electrodes (Scheme 1) was achieved by dipping the functionalized electrode into a 0.2 mM SLAC solution made up in 3 mM sodium phosphate and 33 mM NaCl at pH 7.0, at 4 °C for 4 h. Covalent protein linkage to the carboxyl-terminated SAM (Scheme 1) was made using N-hydroxysuccinimide+1-Ethyl-3-(3-dimethylaminopropyl)carbodiimide (NHS-EDC) [77,78,79]: the MUA- or MUA/MU-modified gold surface was first activated with 200 mM EDC and 50 mM NHS for 5 min, and then transferred into 0.2 mM SLAC for 1 hr. The CVs were carried out using working solutions containing 5 mM Tris-HCl buffer plus 5 mM sodium perchlorate as base electrolyte at pH 7.0. The formal potentials E°′ were calculated as the semi-sum of the anodic and cathodic peak potentials and were almost independent of scan rate in the range 0.02–0.5 Vs^−1^. The experiments were repeated at least three times and the E°′ values were found to be reproducible within ±0.002 V. The CV experiments at different temperatures were carried out with a “non-isothermal” cell in which the reference electrode was kept at a constant temperature (21 ± 0.1 °C) in a 1 M NaClO_4_/Agar salt bridge while the half-cell containing the working electrode and the Vycor^®^ (PAR) junction to the reference electrode was under thermostatic control with a water bath. The temperature was varied from 5 to 45 °C.

With this experimental configuration, the standard entropy change (∆S°′_rc_) is given by:

(5) $$\Delta S{}^{\circ}\prime_{rc}=S{}^{\circ}\prime_{red}-S{}^{\circ}\prime_{ox}=nF\left(\frac{dE{}^{\circ}\prime }{dT}\right)$$

thus, ∆S°′_rc_ was determined from the slope of the plot of E°′ versus temperature which turns out be linear under the assumption that ∆S°′_rc_ is constant over the limited temperature range that was investigated [80,81]. The enthalpy change (∆H°’_rc_) was obtained from the Gibbs–Helmholtz equation, namely as the negative slope of the E°′/T versus 1/T plot. Repeated cycling does not affect the voltammograms from 5 to 45 °C, indicating that the protein monolayer is stable. The non-isothermal behavior of the cell was carefully checked by determining the ∆H°’_rc_ and ∆S°′_rc_ values of the ferricyanide/ferrocyanide couple [65,82,83]. The peak current for the immobilized protein turned out to be linear with the scan rate, as expected for a diffusionless electrochemical process. The surface coverage *Γ*_0_ for the immobilized electrochemically active protein was calculated from the overall charge *Q_tot_* exchanged by the protein (determined upon integration of the baseline-corrected cathodic peaks) and the area *A* of the gold electrode by applying the relationship:

*∫i*(*E*) *dE* = *ν*(*nFAΓ*_0_) = *νQ_tot_* (6)

where *ν* is the scan rate (in Vs^−1^), *n* (=1) the number of electrons exchanged in the redox reaction, and *F* is the Faraday constant. The area of the electrode was determined electrochemically using the Randles–Sevçik equation to the reduction peak of ferrocenium tetrafluoborate of known concentration in aqueous solution, in which the bare electrode was dipped at exactly the same depth as for the measurements with the adsorbed protein. Cyclic voltammograms at different scan rates (from 0.02 to 0.5 Vs^−1^) were recorded to determine the rate constant *k_s_* for the ET process of the adsorbed protein, following the Laviron model for diffusionless electrochemical systems [63]. The effects of uncompensated cell resistance were minimized using the positive-feedback *iR* compensation function of the potentiostat, set at a value slightly below that at which current oscillations emerge [84,85]. The separation between the anodic and the cathodic peak increases with increasing the scan rate, while the E°′ values are unchanged. The *k*_s_ values were averaged over five measurements and found to be reproducible at least within ±1 s^−1^, which was taken as the associate error. The *k_s_* values were measured in the range 5–45 °C to determine the activation enthalpies (ΔH^#^) using the Arrhenius equation, namely from the slope of the plot of ln *k_s_* versus 1/T. Calculation of electrostatic potential of the SLAC surface was carried out with NOC 3.01 software (http://noch.sourceforge.net/, accessed on 20 September 2022).

## 4. Conclusions

Care must be taken with the thermodynamic and kinetic features of the ET for electroactive proteins immobilized on variably functionalized solid electrodes. The latter in fact constitute the core components of bioelectronic sensing devices, fuel cells, and field-effect biotransistors, and the above parameters determine how the protein does its job and talks to the electrode under certain working conditions. Multi-copper oxidases are widely used as cathodes for dioxygen reduction in biofuel cells and in biosensors. Here, we found that the thermodynamics and kinetics of ET of SLAC, a bacterial laccase with a relatively low molecular weight and an efficient expression system in *E. coli*, hence a large practical exploitability, are remarkably sensitive to the electrostatics, pH-induced protein changes, and solvent reorganization effects at the SAM-SLAC interface. Most of these effects, included the affinity of the protein with the functionalized electrode surface, can be interpreted on an electrostatic basis. Although this variability may constitute a drawback in terms of catalytic activity, which is affected by the protein E°′ value and the kinetic of protein–electrode ET, it can also constitute a resource in terms of ability to modulate these parameters on rational bases to obtain a certain bioeffect. In this vein, this paper adds further elements to enable the prediction of immobilization-induced changes in the catalytic or reaction properties of a protein placed into an electrochemical environment for practical purposes.

## Figures and Tables

**Table 1:** Midpoint potential (E°′), surface coverage values (Γ_0_), and reduction thermodynamic parameters for *Streptomyces coelicolor* small laccase (SLAC) immobilized on different functionalized gold electrodes ^a^.

	E°′ (V) (vs. SHE) ^b^	Γ_0_ (pmol cm^−2^) ^b^	ΔH°’_rc_ (kJ/mol)	ΔS°′_rc_ (J/mol K)
MUA	+0.097	1.00	−33.1	−80
3:1 MUA/MU	+0.140	0.71	−38.8	−86
1:1 MUA/MU	+0.158	0.32	−46.7	−107
1:2 MUA/MU	+0.199	0.15	−60.1	−138
MUA (EDC/NHS)	+0.086	4.63	−32.7	−83
3:1 MUA/MU (EDC/NHS), pH 7.0	+0.115	1.22	−39.8	−98
1:2 MUA/MU (EDC/NHS), pH 7.0	+0.145	0.49	−52.1	−128
1:1 MUA/MU (EDC/NHS), pH 7.0	+0.126	1.51	−45.5	−114
1:1 MUA/MU (EDC/NHS), pH 8.4	+0.057	1.43	−47.0	−141
1:1 MUA/MU (EDC/NHS), pH 9.6	−0.021	1.58	−44.2	−157

^a^ At pH = 7.0, unless otherwise specified. Errors affecting E°′, Γ_0_, ΔS^0^’_rc_, and ΔH^0^’_rc_ are ±0.002 V, ±10%, ±2 J/mol K, and ±0.4 kJ/mol, respectively. Working solution: 5 mM Tris-HCl buffer plus 5 mM sodium perchlorate. ^b^ 20 °C.

**Table 2:** Reduction thermodynamics for *Streptomyces coelicolor* small laccase (SLAC) and other immobilized blue-copper proteins at neutral pH.

Protein	E°′ (V) (vs. SHE) ^a^	ΔS°′_rc_ (J/mol K)	ΔH°’_rc_ (kJ/mol)	Immobilization	Ref
SLAC (*S. coelicolor*)	+0.126	−114	−45.5	covalent 1:1 MUA:MU	this work
SLAC (*S. coelicolor*)	+0.158	−107	−46.7	electrostatic 1:1 MUA:MU	this work
Plastocyanin (*S. oleracea*)	+0.090	−61	−26.8	electrostatic 1:1 MUA:MU	[54]
Plastocyanin (*P. laminosum*)	+0.369	−65	−54	cysteamine	[59]
*Synechocystis* sp. PCC 6803	+0.365	−41	−47	cysteamine	[59]
Azurin (*P. aeruginosa*)	+0.272	−14	−30.4	Decane-1-thiol	[60]
Stellacyanin (*Rhus vernicifera*)	+0.143	−54	−29.6	decane-1-thiol	[57]
Laccase McoG (*A. niger*)	+0.453	−28	−51.9	decane-1-thiol	[61]
Cytochrome c oxidase Cu_A_	+0.261	−42	−38	6-mercapto-1-hexanol	[62]

^a^ 20 °C.

**Table 3:** Kinetic constants, *k_s_* (s^−1^), for immobilized *Streptomyces coelicolor* small laccase (SLAC) at selected pH values and temperatures, together with activation enthalpies and reorganization energies for heterogeneous ET between the immobilized enzyme and the electrode.

	*k_s_* (5 °C)	*k_s_* (15 °C)	*k_s_* (25 °C)	ΔH^#^	λ	Lower-Upper Tunneling Distance
	s^−1^	s^−1^	s^−1^	kJ/mol	eV	Å
pH 7.0 ^a^	8	10	11	9.1	0.361	20.3–23.3,
pH 8.4 ^a^	14	15	17	6.8	0.270	20.8–23.8
pH 9.6 ^a^	7	9	10	10.4	0.413	19.9–22.9
pH 7.0 ^b^	9	10	12	9.7	0.385	20.1–23.1

^a^ Values obtained for SLAC covalently bound to 1:1 MUA/MU gold electrode through EDC/NHS linkage procedure. Working solution: 5 mM Tris-HCl buffer plus 5 mM sodium perchlorate. Errors affecting *k_s_*, ΔH^#^ and λ are ±1, ±0.3 kJ/mol and ±0.012 eV, respectively. ^b^ Values obtained for SLAC electrostatically immobilized on 1:1 MUA/MU gold electrode.

## Data Availability

The data presented in this study are available on request from the corresponding author.

## Sample Availability

Samples of the compounds are not available from the authors.

## Abbreviations

SLAC: Small Laccase for *Streptomyces coelicolor*. BMCOs: Multicopper blue oxidases.
